# Antibiotic Use in Late Preterm and Full-Term Newborns

**DOI:** 10.1001/jamanetworkopen.2024.3362

**Published:** 2024-03-22

**Authors:** Johan Gyllensvärd, Marie Studahl, Lars Gustavsson, Elisabet Hentz, Karin Åkesson, Huiqi Li, Mikael Norman, Anders Elfvin

**Affiliations:** 1 Department of Pediatrics, Ryhov County Hospital, Jönköping, Sweden; 2 Department of Pediatrics, Institution of Clinical Sciences, Sahlgrenska Academy, University of Gothenburg, Gothenburg, Sweden; 3 Department of Infectious Diseases, Institute of Biomedicine, Sahlgrenska Academy, University of Gothenburg, Gothenburg, Sweden; 4 Department of Infectious Diseases, Region Västra Götaland, Sahlgrenska University Hospital, Gothenburg, Sweden; 5 Department of Pediatrics, Region Västra Götaland, The Queen Silvia Children’s Hospital, Sahlgrenska University Hospital, Gothenburg, Sweden; 6 Division of Children’s and Women’s Health, Department of Clinical and Experimental Medicine, Linköping University, Linköping, Sweden; 7 School of Public Health and Community Medicine, Institute of Medicine, Sahlgrenska Academy, University of Gothenburg, Gothenburg, Sweden; 8 Department of Clinical Science, Intervention, and Technology, Karolinska Institutet, Stockholm, Sweden; 9 Department of Neonatal Medicine, Karolinska University Hospital, Stockholm, Sweden

## Abstract

**Importance:**

Antibiotic treatment saves lives in newborns with early-onset sepsis (EOS), but unwarranted antibiotic use is associated with resistant bacteria and adverse outcomes later in life. Surveillance is needed to optimize treatment strategies.

**Objective:**

To describe antibiotic use in association with the incidence and mortality from EOS among late-preterm and full-term newborns.

**Design, Setting, and Participants:**

The Sweden Neonatal Antibiotic Use study was a nationwide observational study that included all late-preterm and full-term neonates born from January 1, 2012, to December 31, 2020, in neonatal units of all levels. All hospital live births from 34 weeks’ gestation during the study period were included in the study. Data were collected from the Swedish Neonatal Quality Register and the Swedish Medical Birth Register. Data were analyzed from August 2022 to May 2023.

**Exposure:**

Admission for neonatal intensive care during the first week of life.

**Main Outcomes and Measures:**

The main outcomes were the usage of intravenous antibiotics during the first week of life, the duration of antibiotic therapy, the rate of culture-proven EOS, and mortality associated with EOS.

**Results:**

A total of 1 025 515 newborns were included in the study; 19 286 neonates (1.88%; 7686 girls [39.9%]; median [IQR] gestational age, 40 [38-41] weeks; median [IQR] birth weight, 3610 [3140-4030] g) received antibiotics during the first week of life, of whom 647 (3.4%) had EOS. The median (IQR) duration of antibiotic treatment in newborns without EOS was 5 (3-7) days, and there were 113 antibiotic-days per 1000 live births. During the study period there was no significant change in the exposure to neonatal antibiotics or antibiotic-days per 1000 live births. The incidence of EOS was 0.63 per 1000 live births, with a significant decrease from 0.74 in 2012 to 0.34 in 2020. Mortality associated with EOS was 1.39% (9 of 647 newborns) and did not change significantly over time. For each newborn with EOS, antibiotic treatment was initiated in 29 newborns and 173 antibiotic-days were dispensed.

**Conclusions and Relevance:**

This large nationwide study found that a relatively low exposure to antibiotics is not associated with an increased risk of EOS or associated mortality. Still, future efforts to reduce unwarranted neonatal antibiotic use are needed.

## Key Points

**Question:**

What is the proportion of antibiotic use and the incidence and deaths from early-onset sepsis (EOS) among late-preterm and full-term newborns?

**Findings:**

This nationwide cross-sectional study included more than 1 million newborns in Sweden. Of these, nearly 20 000 were treated with antibiotics during the first week of life, 647 had EOS, and 9 newborns with EOS died.

**Meaning:**

These findings suggest that a large number of newborns are treated with antibiotics without having EOS and that there is a potential to reduce antibiotic use.

## Teaser

This cross-sectional study examines antibiotic use in association with the incidence and mortality from early-onset sepsis among late-preterm and full-term newborns in Sweden.

## Introduction

Antibiotic use in the neonatal period has been reported to vary between 1.2% and 14.0% among late-preterm and full-term neonates.^1,2,3,4^ Antibiotic overuse is associated with increased risk of colonization with antibiotic-resistant bacteria.^5^ To avoid an antimicrobial resistance crisis, the World Health Organization has called for urgent action promoting antimicrobial stewardship programs to help clinicians improve antibiotic prescription practices and patients’ outcomes.^6^

Neonatal sepsis is a serious condition that could rapidly become life-threatening; hence, clinical suspicion often leads to antibiotic therapy in newborns.^7^ Fear of missing sepsis in addition to suboptimal diagnostic biomarkers contribute to antibiotics being the most prescribed drug in neonatal units.^8,9,10,11,12^ Antibiotic exposure in early life influences the developing microbiome and may be associated with an increased risk of asthma, allergy, overweight, diabetes, and inflammatory bowel disease later in life.^13,14^ Moreover, neonatal antibiotic use is associated with longer hospital stay and increased health care costs.^15,16^ Therefore, it is paramount to reduce unwarranted antibiotic use in newborns.

One way of addressing the magnitude of unwarranted treatment would be to study changes over time in neonatal antibiotic use in relation to early-onset sepsis (EOS) and mortality. Longitudinal monitoring of antibiotic use in relation to EOS and mortality is needed to optimize treatment strategies. The aim of this nationwide, population-based cohort study of late-preterm and full-term newborns was to assess antibiotic treatment, incidence of culture-positive EOS, and all-cause and EOS-associated mortality from 2012 to 2020. An additional aim was to compare outcomes at different levels of care to assess any potential differences and to facilitate benchmarking.

## Methods

### Study Design, Setting, and Population

**Figure 1.**
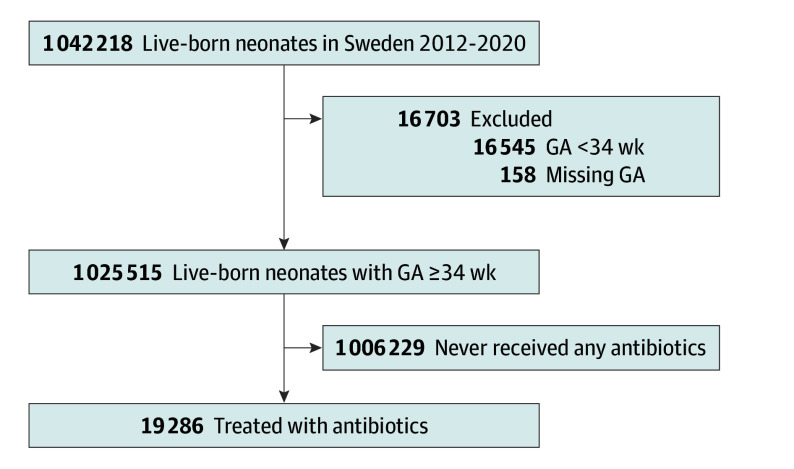
Newborns Enrolled in the Study GA indicates gestational age.

The Sweden Neonatal Antibiotic Use (SWENAB) study is a nationwide observational study that included all liveborn newborns with a gestational age (GA) greater than or equal to 34 weeks born in Sweden during the 9-year period from January 1, 2012, to December 31, 2020. Newborns with GA less than 34 weeks or unknown GA were excluded (Figure 1). The Swedish Ethical Review Authority approved the study and granted a waiver of informed consent. The present study followed the Strengthening the Reporting of Observational Studies in Epidemiology for Newborn Infection (STROBE-NI) reporting guidelines.^17^

Sweden has 10.5 million inhabitants, 44 obstetric departments, and 37 neonatal units, treating preterm and full-term newborns who need medical care including intravenous antibiotics. Three designated levels of neonatal intensive care units (NICUs) (level I, level II, and level III) generally correspond to American Academy of Pediatrics levels I to II, II to III, and III to IV, respectively.^18^ Level I (7 units) and level II (22 units) units were able to provide risk-appropriate care of neonates more than 31 weeks’ GA (2 units cared for neonates more than 33 and 34 weeks’ gestation at birth, respectively) and 27 weeks’ GA at birth, respectively. Level III units (8 units) were university hospitals specialized in caring for the sickest and most preterm newborns, and 4 of these 8 units can also provide surgical repair of complex congenital or acquired conditions and correspond to American Academy of Pediatrics level IV.

In Sweden in 2020, neonatal death rate and stillbirth rates were 1.7 and 3.1 per 1000 live births, respectively, and 5.4% of neonates were born preterm (<37 weeks).^19^ The rate of home births in Sweden was 1‰.^20^ Health care services during pregnancy and childhood were free of charge for all inhabitants.

There was no general screening in Sweden for group B *Streptococcus* (GBS) bacteriuria or rectovaginal colonization in pregnant women without symptoms during the study period. The national strategy for prevention of early neonatal GBS sepsis was a risk-based approach implemented in 2008.^21^ Intrapartum prophylactic antibiotics were given to pregnant individuals if they had GBS bacteriuria, fever during delivery, a previous newborn with GBS sepsis, or rupture of membranes lasting longer than 18 hours. There was no change in national guidelines regarding the management of chorioamnionitis during the study period. Furthermore, there was no national guideline on when to start or discontinue antibiotic treatment in neonates. National antimicrobial guidelines issued in 2013 suggested the use of intravenous benzylpenicillin and aminoglycoside in suspected EOS of the newborn.^22^

### Data Sources

Data were obtained from the Swedish Neonatal Quality Register (SNQ), including all neonatal care admissions in Sweden since 2011.^23^ Data including diagnosis, interventions, and treatment were reported to SNQ by the attending staff. Diagnoses were classified according to *International Statistical Classification of Diseases and Related Health Problems, Tenth Revision* codes.^24^ The total number of live births was collected from the National Medical Birth Registry held by the National Board of Health and Welfare.^25^

### Study Definitions

Culture-positive sepsis was defined as growth of bacteria in blood cultures or cerebrospinal fluid, clinical signs and symptoms compatible with sepsis, elevated biomarkers for infection according to the unit’s guidelines, and intravenous antibiotic treatment for at least 5 days (or less if the patient died before 5 days of treatment or was transferred to a non-NICU health care facility). Coagulase-negative staphylococci (CoNS) and *Bacillus* or *Micrococcus* species grown in blood cultures taken the first week of life were considered as contaminations and cultures with fungi (1 patient with *Candida* species) were excluded in this study of full-term and late-preterm newborns.^26^

EOS was defined as culture-positive sepsis within 3 days of birth (days 0-2). Sepsis in the first week of life was defined as culture-positive sepsis within 7 days of life (days 0-6). Rates of EOS were calculated per 1000 live births. EOS-associated mortality was defined as newborns with EOS who died, and mortality was defined as death within 28 days of age.

Neonates treated with antibiotics were classified as having culture-positive sepsis or no sepsis. In SNQ, culture-positive sepsis was registered by the attending physician by *International Statistical Classification of Diseases and Related Health Problems, Tenth Revision* codes P36.0 to P36.8, A40.0, A40.1, A41.0, A41.1, and G00.0 to G00.8 or by reporting the diagnosis using checkboxes and free text. No sepsis was defined as neonates treated with antibiotics but not classified as having culture-positive sepsis. In contradicting cases that were difficult to classify, the final classification was determined by a consensus judgment by the author group.

### Outcomes

The primary outcome was the use of intravenous antibiotics during the first week of life. Secondary outcomes were the duration of antibiotic treatment in days, the incidence of culture-proven sepsis in the first week of life, the incidence of EOS, and EOS-associated mortality. Additional outcomes were types of pathogens in culture-positive sepsis. To assess any potential variations between different levels of care and to facilitate benchmarking, we present data on EOS and antibiotic use stratified by levels of care and considering different case mixes, as proposed by Giannoni et al.^1^

### Statistical Analysis

Median (IQR) and numbers (percentages) were calculated. Trends over time were assessed with Jonkheere-Terpstra test for continuous variables and Mantel-Haenszel test for categorial variables. Statistical significance refers to *P* < .05 (2-sided). All analyses were performed using SPSS statistical software version 29 (IBM). Data were analyzed from August 2022 to May 2023.

## Results

**Figure 2.**
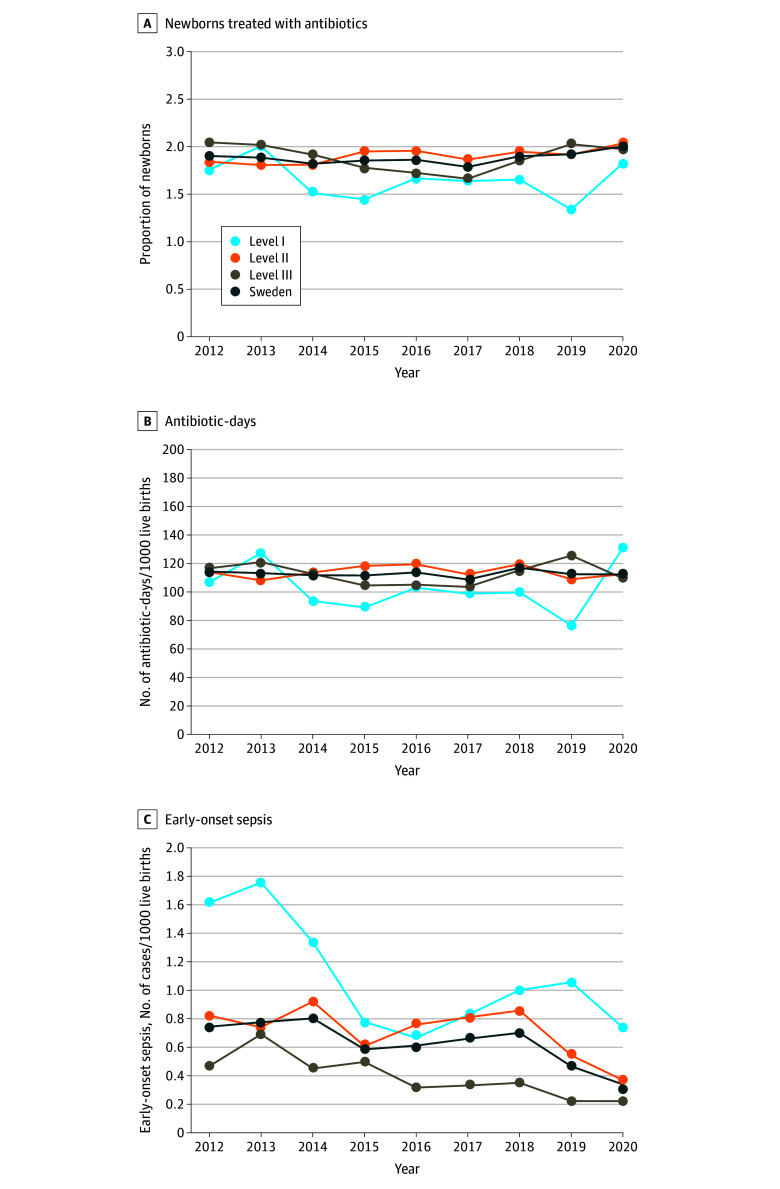
Antibiotic Use and Early-Onset Sepsis A, Percentages of late-preterm and full-term newborns treated with antibiotics in different levels of care and total in Sweden. B, Number of antibiotic-days per 1000 live births in late-preterm and full-term newborns in different levels of care and total in Sweden. C, Late-preterm and full-term newborns with early-onset sepsis, rate per 1000 live births by year in different levels of care and total in Sweden.

The total cohort consisted of 1 025 515 newborns born at GA 34 weeks or later, of whom 19 286 (1.88%; 7686 girls [39.9%]; median [IQR] gestational age, 40 [38-41] weeks; median [IQR] birth weight, 3610 [3140-4030] g) had been treated with antibiotics in the first week of life (Figure 1 and Table 1). There were 984 229 full-term newborns (96% of the cohort), of whom 16 572 (1.68%) were treated with antibiotics. The proportion of newborns treated with antibiotics more than 3 days was 1.39%, and 0.89% of newborns were treated for more than 5 days (eFigure in Supplement 1). During the study period, there was no statistically significant change in the proportion of newborns treated with antibiotics (Table 2 and Figure 2). The median (IQR) duration of antibiotic treatment was 8 (7-10) days for newborns with EOS and 5 (3-7) days for those without sepsis (Table 1). There were 113 antibiotic-days per 1000 live births.

**Table 1.** Clinical and Demographic Characteristics of Newborns Treated With Antibiotics During the First Week of Life^a^

Clinical characteristics	Newborns, No. (%)			
	All (N = 19 286)	Culture-positive sepsis (n = 719)	Early-onset sepsis (n = 647)	No sepsis (n = 18 567)
Sex^b^				
Female	7686 (39.9)	274 (38.1)	256 (39.6)	7412 (39.9)
Male	11 598 (60.1)	445 (61.9)	391 (60.4)	11 153 (60.1)
Gestational age, median (IQR), wk	40 (38-41)	40 (38-41)	40 (38-41)	40 (38-41)
Birth weight, median (IQR), g^c^	3610 (3140-4030)	3610 (3160-4010)	3625 (3195-4020)	3610 (3140-4040)
Small for gestational age^d^	524 (2.7)	19 (2.6)	13 (2.0)	505 (2.7)
Large for gestational age^e^	1039 (5.4)	22 (3.1)	18 (2.8)	1017 (5.5)
Apgar score at 5 min <7^f^	3346 (17.6)	80 (11.3)	75 (11.7)	3266 (17.9)
Apgar score at 10 min <7^g^	1749 (9.2)	27 (3.8)	26 (4.1)	1722 (9.4)
Rupture of membranes^h^				
0-12 h before birth	12 064 (69.5)	434 (65.8)	384 (64.5)	11 630 (69.6)
12-24 h before birth	2666 (15.4)	121 (18.3)	112 (18.8)	2545 (15.2)
>24 h before birth	2638 (15.2)	105 (15.9)	99 (16.6)	2533 (15.2)
Chorioamnionitis^i^	189 (1.0)	14 (2.1)	13 (2.2)	175 (1.5)
Duration of treatment, median (IQR), d	5 (3-7)	9 (7-10)	8 (7-10)	5 (3-7)
Umbilical vein catheter	4841 (25.1)	143 (19.9)	126 (19.5)	4698 (25.3)
Umbilical artery catheter	3478 (18.0)	104 (14.5)	90 (13.9)	3374 (18.2)
Central vein catheter	1001 (5.2)	52 (7.2)	40 (6.2)	949 (5.1)
Ventilator	2595 (13.5)	56 (7.8)	49 (7.6)	2539 (13.7)
Hospital stay, median (IQR), d	8 (5-11)	10 (8-12)	9 (8-12)	7 (5-11)
Mortality within 28 d	276 (1.43)	10 (1.39)	9 (1.39)	266 (1.43)

^a^ Categorial and continuous variables are displayed as numbers (percentages), and as median (IQR) values. Early-onset sepsis was defined as culture-positive sepsis within three days of birth (day 0-2).

^b^ Data are missing for 2 neonates each in the all treated and no sepsis groups.

^c^ Data are missing for 280 neonates in the all treated group, 6 in the culture-positive sepsis group, 6 in the early-onset sepsis group, and 274 in the no sepsis group.

^d^ Small for gestational age was defined as birth weight below 2 SD from the mean.

^e^ Large for gestational age was defined as birth weight more than 2 SD from the mean.

^f^ Data are missing for 290 neonates in the all treated group, 9 in the culture-positive sepsis group, 6 in the early-onset sepsis, and 281 in the no sepsis group.

^g^ Data are missing for 315 neonates in the all treated group, 9 in the culture-positive sepsis group, 7 in the early-onset sepsis group, and 306 in the no sepsis group.

^h^ Data are missing for 1918 neonates in the all treated group, 59 in the culture-positive sepsis group, 52 in the early-onset sepsis group, and 1859 in the no sepsis group.

^i^ Data are missing for 840 neonates in the all treated group, 62 in the culture-positive sepsis group, 59 in the early-onset sepsis group, and 778 in the no sepsis group.

**Table 2.** Main Outcomes Among Newborns Treated With Antibiotics During the First Week of Life, 2012 to 2020^a^

Outcomes	2012 (n = 110 213)	2013 (n = 110 837)	2014 (n = 113 357)	2015 (n = 114 531)	2016 (n = 119 394)	2017 (n = 115 311)	2018 (n = 115 548)	2019 (n = 113 922)	2020 (n = 112 402)	*P* value^b^
Treated newborns by GA, No. (%)										
≥34 wk	2096 (1.90)	2089 (1.89)	2063 (1.82)	2123 (1.86)	2223 (1.86)	2060 (1.79)	2194 (1.90)	2187 (1.92)	2251 (2.00)	.06
≥37 wk	1774 (1.7)	1795 (1.7)	1770 (1.6)	1826 (1.7)	1929 (1.7)	1765 (1.6)	1896 (1.7)	1859 (1.7)	1958 (1.8)	.02
34-36 wk	322 (7.1)	294 (6.5)	293 (6.3)	297 (6.4)	294 (6.1)	295 (6.4)	298 (6.5)	328 (7.3)	293 (6.7)	.61
Duration of antibiotic treatment, median (IQR), d										
All treated newborns	5 (3-7)	5 (3-7)	6 (3-7)	5 (3-7)	5 (3-7)	5 (3-7)	5 (4-7)	5 (3-7)	5 (3-7)	<.001
EOS^c^	8 (7-10)	10 (7-10)	8 (7-10)	7 (7-11)	9 (7-10)	9 (7-10)	9 (7-10)	8 (7-10)	8 (7-10)	.59
No sepsis	5 (3-7)	5 (3-7)	6 (3-7)	5 (3-7)	5 (3-7)	5 (3-7)	5 (4-7)	5 (3-7)	5 (3-7)	<.001
Antibiotic-days, No.	12 597	12 550	12 670	12 780	13 574	12 539	13 521	12 814	12 623	NA
Antibiotic-days/1000 live births, No.	114	113	112	112	114	109	117	112	112	.82
Rate of newborns with diagnosis, No. (‰)										
EOS (0-2 d)	82 (0.74)	86 (0.78)	91 (0.80)	67 (0.58)	73 (0.61)	76 (0.66)	81 (0.70)	53 (0.47)	38 (0.34)	<.001
Sepsis first wk of life (0-6 d)	89 (0.81)	98 (0.88)	98 (0.86)	73 (0.64)	82 (0.69)	89 (0.77)	89 (0.77)	57 (0.50)	44 (0.39)	<.001
No sepsis	2007 (18.2)	1991 (18.0)	1965 (17.3)	2050 (17.9)	2141 (17.9)	1971 (17.1)	2105 (18.2)	2130 (18.7)	2207 (19.6)	<.001
All-cause deaths, No. (%)										
All newborns^d^	47 (0.043)	75 (0.068)	46 (0.041)	52 (0.045)	55 (0.046)	48 (0.042)	51 (0.044)	43 (0.038)	51 (0.045)	.13
All antibiotic-treated newborns	26 (1.2)	35 (1.7)	28 (1.4)	34 (1.6)	31 (1.4)	23 (1.1)	36 (1.6)	29 (1.3)	34 (1.5)	.76
EOS	1 (1.2)	3 (3.5)	1 (1.1)	0	1 (1.4)	1 (1.3)	1 (1.2)	0	1 (2.6)	.29
No sepsis	25 (1.2)	31 (1.5)	27 (1.3)	34 (1.6)	30 (1.3)	22 (1.1)	35 (1.6)	29 (1.3)	33 (1.5)	.56

Abbreviations: EOS, early-onset sepsis; GA, gestational age; NA, not applicable.

^a^ Categorial and continuous variables are displayed as frequencies (percentages), and as median (IQR), respectively.

^b^ *P* value calculated with trend test.

^c^ EOS was defined as culture-positive sepsis within 3 days of birth (day 0-2).

^d^ All newborns include all live births during the study period and not only the newborns treated with antibiotics during the first week of life.

### Incidence of Sepsis

The incidence of EOS (days 0-2) was 0.63 per 1000 newborns (647 of 1 025 515 newborns) and that of culture-proven sepsis in the first week of life (days 0-6) was 0.70 per 1000 newborns (719 of 1 025 515 newborns). Among neonates treated with antibiotics, the rate of EOS was 3.4% (647 neonates). EOS and culture-proven sepsis during the first week of life decreased throughout the study period from 0.74 per 1000 newborns in 2012 to 0.34 per 1000 newborns in 2020 for EOS (Table 2 and Table 3).

**Table 3.** Change in Rates of Early-Onset Sepsis Over 2 Time Periods^a^

Variable	No. of cases/total No. (‰)		*P* value^b^
	Period 1 (2012-2014)	Period 2 (2018-2020)	
All	259/334 407 (0.77)	172/341 872 (0.50)	<.001
GA ≥37 wk	233/320 692 (0.73)	156/328 448 (0.47)	<.001
GA 34-36 wk	26/13 709 (1.90)	16/13 694 (1.17)	.16
Group B *Streptococcus*	155/334 407 (0.46)	86/341 872 (0.25)	<.001
GA ≥37 wk	143/320 692 (0.46)	79/328 448 (0.24)	<.001
GA 34-36 wk	12/12 709 (0.88)	7/13 694 (0.51)	.36
*Escherichia coli*	23/334 407 (0.07)	19/341 872 (0.06)	.54
GA ≥37 wk	21/320 698 (0.07)	17/328 448 (0.05)	.52
GA 34-36 wk	2/13 709 (0.15)	2/13 694 (0.15)	>.99

Abbreviation: GA, gestational age.

^a^ Pathogens are displayed as frequencies (per thousand) and are categorized by gestational weeks and pathogens. Early-onset sepsis was defined as culture-positive sepsis within 3 days of birth (days 0-2).

^b^ *P* values were calculated with χ^2^ test.

The number of positive cultures, both regarding gram-positive and gram-negative pathogens, during the first week of life decreased by more than 50% from 2012 to 2020 (eTable 1 in Supplement 1). GBS (365 of 719 cultures [51%]), *Escherichia coli* (81 of 719 cultures [11%]), and *Staphylococcus aureus* (74 of 719 cultures [10%]) were the most common bacteria found in blood cultures (eTable 1 in Supplement 1). For each newborn with EOS, antibiotic treatment was initiated in 29 newborns and 173 antibiotic-days were dispensed. During the study period, there was an increase in the number of newborns treated with antibiotics per culture-positive EOS case (days 0-2) and per culture-positive sepsis case during the first week (days 0-6), as well as in the number of antibiotic-days per sepsis case and in the number of no sepsis cases per proven sepsis case, with a pronounced increase in the last 2 years (eTable 2 in Supplement 1).

### Mortality

The number of deaths among all live births in the study population was 468, and the all-cause mortality rate was 0.46 per 1000 live births. The mortality rate among newborns treated with antibiotics was 1.43% (276 of 19 286 newborns), was distributed fairly evenly between those with EOS (9 of 647 newborns [1.39%]) and those without sepsis (266 of 18 567 newborns [1.44%]), and did not change significantly over time (Table 1 and Table 2). Among the 9 newborns with EOS who died within 28 days of age, 6 deaths were classified as being caused by sepsis, whereas the remaining 3 had other causes of death associated with complications after perinatal asphyxia. Among the 6 newborns who died of EOS, 5 died within 4 days (3 cases of *E coli*, 1 case of GBS, and 1 caused by other bacteria) and 1 died within 9 days of age (*E coli*). In cases with EOS, the mortality rate was 1.03% (6 of 583 newborns) among full-term newborns and 4.69% (3 of 64 newborns) among newborns with GA of 34 to 36 weeks. EOS caused 1.92% (9 of 468 newborns) of all-cause deaths. In total, 1 of 113 946 newborns died of EOS (9 of 1 025 515 newborns [0.0088‰]).

### Levels of Care

Antibiotic use rates were 1.63% in level I units (964 of 59 100 newborns), 1.90% in level II units (12 128 of 637 189 newborns), and 1.89% in level III units (6194 of 328 246 newborns). The number of antibiotic-days per 1000 live births was 102 in level I units, 114 in level II units, and 113 days in level III units. The percentage of newborns exposed to antibiotics during the first week of life and antibiotic-days per 1000 live births fluctuated over time, and the largest variations were in level I units (Figure 2). The incidence of EOS was 1.1 per 1000 live births (63 of 59 100 newborns) in level I units, 0.71 per 1000 live births (455 of 637 189 newborns) in level II units, and 0.39 per 1000 live births (129 of 328 246 newborns) in level III units (Figure 2). The number of deaths was 18 in level I units, 234 in level II units, and 216 in level III units; all-cause mortality was 0.30 per 1000 live births in level I units, 0.37 per 1000 live births in level II units, and 0.66 per 1000 live births in level III units. EOS-associated mortality was 1.6% (1 of 63 newborns) in level I units, 1.1% (5 of 455 newborns) in level II units, and 2.3% (3 of 129 newborns) in level III units.

## Discussion

This comprehensive, nationwide cross-sectional study of more than 1 million late-preterm and full-term newborns found low rates of antibiotic use (1.88%) in the first week of life and low risks of EOS and EOS-associated mortality. For each newborn with EOS, antibiotic treatment was initiated in 29 newborns and 173 antibiotic-days were dispensed.

In the present study, the antibiotic use for each case of EOS was considerably lower compared with that in previous studies^3,27,28^; however, the antibiotic use for each case of EOS increased in 2019 and 2020, mainly owing to a decrease in the incidence of culture-positive sepsis without a concomitant reduction in antibiotic use. Consequently, there was an increase in the percentage of newborns treated with antibiotics in the group who had no sepsis during the study period, which is an unwanted evolution that needs to be addressed. Our results indicate that a relatively low rate of antibiotic use among late-preterm and full-term newborns with maintained low morbidity and mortality is possible in a high-income setting. Yet, there remains a high burden of treatment compared with the incidence of EOS and mortality.

To improve patient health and quality of care, it is important to study relevant and meaningful metrics of neonatal antibiotic use.^12,29^ In the current study, there was no significant decrease in antibiotic use during the study period, which contrasts with the findings of another nationwide study from Norway.^3^ However, the rate of antibiotic use in our study was low already in the beginning of the study period.

The median duration of antibiotic treatment in newborns without sepsis was 5 days, which is longer than the duration reported by others.^1,3^ International guidelines^7,30^ suggest discontinuation of antibiotics within 36 to 48 hours if the clinical suspicion of EOS has substantially decreased and the blood cultures remain negative. Our results indicate a potential to reduce the duration of antibiotic treatment, in addition to decreasing the initiation of antibiotic treatment in the group who had no sepsis. In late 2020, national guidelines were published that may help to reduce unnecessary antibiotic use in the future.^31^ The guidelines comprise several recommendations, including not to treat with prophylactic antibiotics when using intravenous catheters and to discontinue antibiotic treatment after 48 to 72 hours if blood cultures remain negative and the clinical suspicion of sepsis has decreased.^31^

The incidence of EOS in high-income countries in late-preterm and full-term newborns varies from 0.13 to 1.45 per 1000 live births and has decreased in recent decades, which is in line with our results.^1,2,27,32,33,34,35^ The significant decrease in EOS during the study period was mainly due to a reduction in the incidence of GBS, which may be due to intrapartum antibiotic prophylaxis guidelines.^36,37,38^ In addition, the EOS-associated mortality rate in the current study was 1.39%, which is lower than reported in several other studies.^1,2,3,32,39^

Considering the diverse case mix and to facilitate benchmarking, we stratified hospitals by different levels of care. As expected, the percentage of newborns exposed to antibiotics and antibiotic-days per 1000 live births was lower in level I units compared with level II and level III units. Surprisingly, level I hospitals had the highest incidence of EOS in the study. There was no recommendation to manage prevention and treatment of EOS differently at level I units, and, therefore, we have no explanation for this finding. Reassuringly, however, EOS-associated mortality was lower in level I and level II units compared with level III units. These results indicate that NICUs with a lower level of care may have an opportunity to decrease antibiotic use even more than presently shown.

There are different strategies to reduce neonatal antibiotic use.^2,9,15,40,41,42^ One of the most popular strategies in recent years is using the EOS calculator.^2^ Several studies^2,4,9^ have reported successful reduction of antibiotic use after implementing the EOS calculator. Serial physical examinations is another strategy, suggested by the American Academy of Pediatrics,^7^ to identify newborns with suspected sepsis and reduce antibiotic use.^40,42,43^ Asymptomatic newborns with risk factors for infection are usually not treated with antibiotics in Sweden. They are followed by clinical examinations, and antibiotic treatment is started only if symptoms of sepsis develop. Antimicrobial stewardship programs aimed at safely reducing administration of unwarranted antibiotic treatment and encouraging safe discontinuation of antibiotics within 36 to 48 hours have also been reported.^41,44,45^

This study presents nationwide population-based data on antibiotic use over time and by level of care in Sweden. In future studies, we plan to explore further the regional variation in antibiotic use and the numbers and proportions of culture-negative sepsis.

### Limitations

Even though our study has a large sample size and a nationwide population-based design, there are identified limitations. The high-income setting may limit the generalizability to other settings. Although the SNQ have predefined diagnostic criteria and alert against unlikely numbers in the web form, inaccuracies in the data reporting may have occurred.^23^ As in all registry-based studies, there is a risk of systematic error in the registered data. However, previous reports have demonstrated that SNQ data are of high quality.^23^ Moreover, in 2016 an online, everyday reporting tool (SNQreg), was implemented.^23^ The everyday reporting minimizes the risk of potential errors in the data. In this data set, there was no information on how antibiotic treatment was administered or the exact time when treatment was started or discontinued. Furthermore, we did not have access to any information on intrapartum antibiotic prophylaxis, clinical symptoms of the newborn, or biomarkers. However, it is reasonable to assume that antibiotic treatment was administered intravenously as praxis and was started the same day the neonate received a diagnosis of infection. In line with previous studies of near-term and full-term newborns, we considered growth of CoNS in the blood culture as contaminants.^3,10,26^ However, we cannot rule out that some of the blood cultures with growth of CoNS represented true sepsis. There is no universal definition of sepsis or a clear boundary between EOS and late-onset infections, making comparisons with other studies difficult. To facilitate such comparisons, we included data on both EOS within 3 days of age (days 0-2) and sepsis within the first week of life (days 0-6).

## Conclusions

This large nationwide study found that relatively low exposure to antibiotics is not associated with an increased risk of EOS or associated mortality in newborns. Still, there may be a potential to reduce antibiotic use more considering the decreasing incidence of EOS over time and the low mortality rate associated with EOS. Future efforts to reduce unwarranted neonatal antibiotic use in Sweden are needed.

## Notes

Supplement 1. **eTable 1.** Pathogens in Blood Cultures Among Newborns With Sepsis During the First Week of Life in Sweden During 2012-2020^a^ **eTable 2.** Number of Infants Treated With Antibiotics per Sepsis Case in Newborns and Relationship Between Diagnosis and Antibiotic Days per Sepsis Case by Year^a^ **eFigure.** Proportion of Infants Treated With Antibiotics

Supplement 2.
SWENAB Study Group Members

Supplement 3.
Data Sharing Statement
