# Supplementary material for: Antibiotic Use in Late Preterm and Full-Term Newborns
Source: JAMA Netw Open. 2024 Mar 22;7(3):e243362. doi: 10.1001/jamanetworkopen.2024.3362 (PMC10960197; doi:10.1001/jamanetworkopen.2024.3362)
Supplement: Supplement 1. — eTable 1. Pathogens in Blood Cultures Among Newborns With Sepsis During the First Week of Life in Sweden During 2012-2020a eTable 2. Number of Infants Treated With Antibiotics per Sepsis Case in Newborns and Relationship Between Diagnosis and Antibiotic Days per Sepsis Case by Yeara eFigure. Proportion of Infants Treated With Antibiotics [file jamanetwopen-e243362-s001.pdf]

Supplemental Online Content

Gyllensvärd J, Studahl M, Gustavsson L, et al; SWENAB Study Group. Antibiotic use in late preterm and full-term newborns. *JAMA Netw Open*. 2024;7(3):e243362. doi:10.1001/jamanetworkopen.2024.3362

eTable 1. Pathogens in Blood Cultures Among Newborns With Sepsis During the First Week of Life in Sweden During 2012-2020[a]

eTable 2. Number of Infants Treated With Antibiotics per Sepsis Case in Newborns and Relationship Between Diagnosis and Antibiotic Days per Sepsis Case by Year[a]

eFigure. Proportion of Infants Treated With Antibiotics

This supplemental material has been provided by the authors to give readers additional information about their work.

© 2024 Gyllensvärd J et al. *JAMA Network Open.*

eTable 1. Pathogens in Blood Cultures Among Newborns With Sepsis During the First Week of Life in Sweden During 2012-2020[a]

Pathogen	2012	2013	2014	2015	2016	2017	2018	2019	2020	Total
Gram-positive	67 (74)	69 (69)	73 (73)	49 (66)	59 (72)	66 (73)	60 (65)	40 (69)	33 (75)	516 (70)
GBS	57 (63)	49 (49)	55 (55)	29 (39)	39 (48)	47 (52)	37 (40)	29 (50)	23 (52)	365 (50)
Enterococcus species	3 (3)	4 (4)	5 (5)	4 (5)	3 (4)	2 (2)	4 (4)	0	1 (2)	26 (4)
Streptococcus species	1 (1)	2 (2)	3 (3)	1 (1)	2 (2)	5 (5)	2 (2)	1 (2)	1 (2)	18 (2)
Streptococcus pneumoniae	0	2 (2)	1 (1)	2 (3)	1 (1)	0	0	0	0	6 (1)
Staphylococcus aureus	4 (4)	10 (10)	7 (7)	6 (8)	14 (17)	9 (10)	11 (12)	8 (14)	5 (11)	74 (10)
Other *staphylococcus* species	1 (1)	0	0	0	0	0	0	0	0	1 (0.1)
Other Gram-positive species	1 (1)	2 (2)	2 (2)	7 (9)	0	3 (3)	6 (7)	2 (3)	3 (7)	26 (4)
Gram-negative	12 (13)	13 (13)	14 (14)	10 (14)	7 (9)	13 (14)	15 (16)	7 (12)	5 (11)	96 (13)
Echerichia coli	10 (11)	12 (12)	12 (12)	7 (9)	4 (5)	12 (13)	14 (15)	5 (9)	5 (11)	81 (11)
Haemophilus species	0	1 (1)	0	1 (1)	0	0	1 (1)	2 (3)	0	5 (1)
Klebsiella species	2 (2)	0	1 (1)	1 (1)	0	0	0	0	0	4 (1)
Enterobacter species	0	0	0	0	1 (1)	0	0	0	0	1 (0.1)
Pseudomonas species	0	0	1 (1)	0	0	0	0	0	0	1 (0.1)
Other Gram-negative species	0	0	0	1 (1)	2 (2)	1 (1)	0	0	0	4 (1)
Other species[b]	12 (13)	18 (18)	13 (13)	15 (20)	16 (20)	12 (13)	17 (18)	11 (19)	6 (14)	120 (16)
All cases[c]	91 (100)	100 (100)	100 (100)	74 (100)	82 (100)	91 (100)	92 (100)	58 (100)	44 (100)	732 (100)

Abbreviations: GBS, group B Streptococcus.

Thirteen infants had growth of different pathogens and were included, hence the total number of newborns with positive blood cultures is 719.

[a] Pathogens are displayed as numbers (percentages)

[b] Other species: Growth of unspecified species

[c] Excluding coagulase negative staphylococcus (CoNS), viruses and fungi. There were in total 163 cases with growth of CoNS in blood cultures. Of these, 145 neonantes (89 %) received ≥5 days of antibiotics.

© 2024 Gyllensvärd J et al. *JAMA Network Open.*

eTable 2. Number of Infants Treated With Antibiotics per Sepsis Case in Newborns and Relationship Between Diagnosis and Antibiotic Days per Sepsis Case by Year[a]

	2012	2013	2014	2015	2016	2017	2018	2019	2020	*P*[c]
Number of infants treated per EOS case	24.7	23.5	21.9	30.8	29.6	26.0	26.2	40.0	57.7	<.001
Number of infants treated per sepsis case	23.6	21.3	21.1	29.1	27.1	23.1	24.7	38.4	51.2	<.001
Number of No sepsis cases[b] per sepsis case	22.6	20.3	20.1	28.1	26.1	22.1	23.7	37.4	50.2	<.001
Antibiotic days per sepsis case	142	128	129	175	166	141	152	225	287	<.001

Abbreviations: EOS, early-onset sepsis

[a] EOS was defined as culture-positive sepsis within three days of birth (day 0-2). Sepsis cases was defined as culture-positive sepsis within the first week of life (day 0-6).

[b] No Sepsis cases was defined as neonates treated with antibiotics within the first week of life (day 0-6) but who were not classified as culture-positive sepsis (day 0-6).

[c] Jonkheere-Terpstra test

© 2024 Gyllensvärd J et al. *JAMA Network Open.*

eFigure. Proportion of Infants Treated With Antibiotics

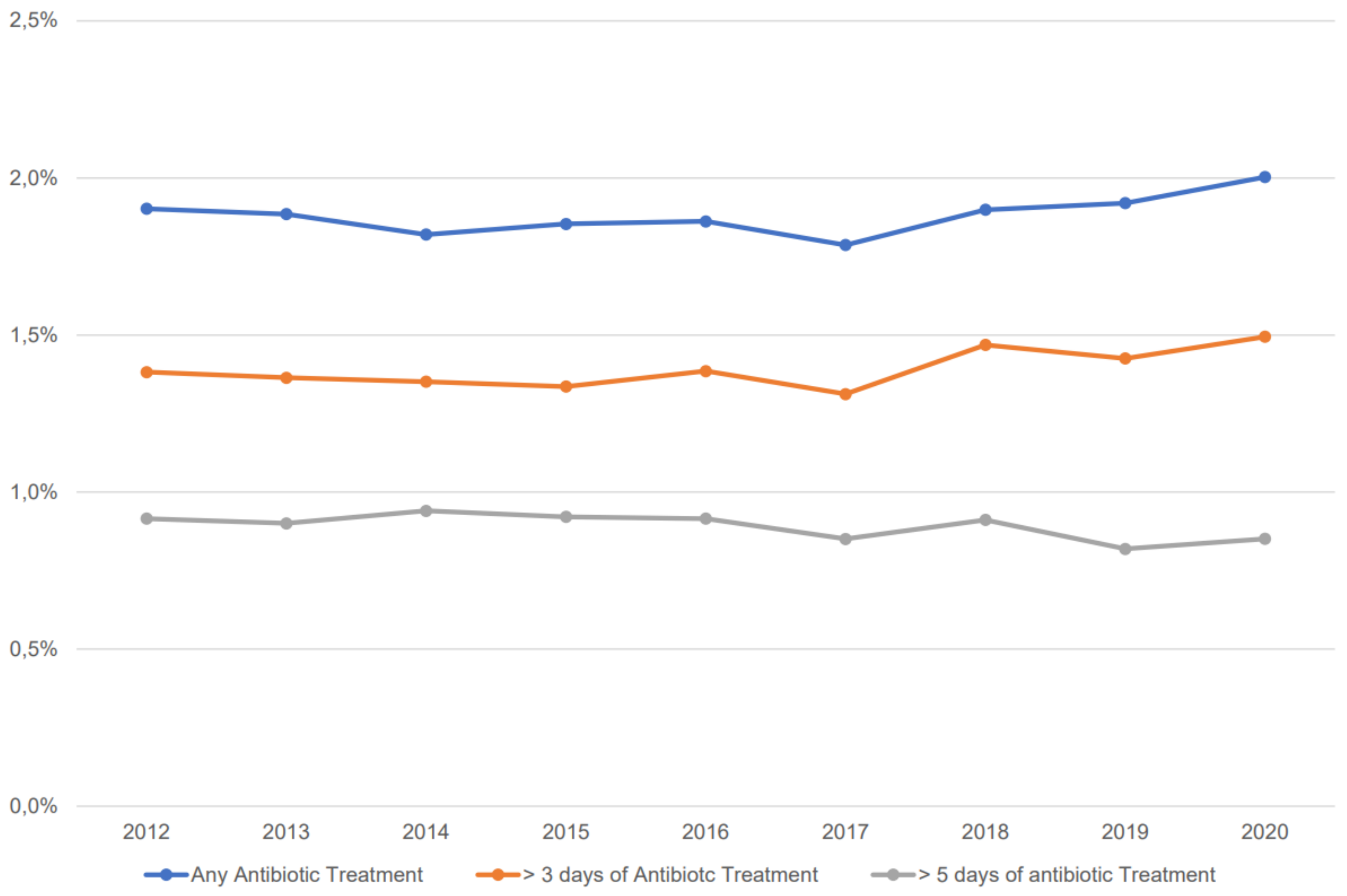

Proportion of infants treated with antibiotics during the first week of life and divided by different durations of treatment.

© 2024 Gyllensvärd J et al. *JAMA Network Open.*
