# Supplementary material for: Antibiotic Use in Late Preterm and Full-Term Newborns
Source: JAMA Netw Open. 2024 Mar 22;7(3):e243362. doi: 10.1001/jamanetworkopen.2024.3362 (PMC10960197; doi:10.1001/jamanetworkopen.2024.3362)
Supplement: Supplement 2. — SWENAB Study Group Members [file jamanetwopen-e243362-s002.pdf]

Supplemental Online Content: Nonauthor Collaborators
*First name, last name, and suffix (if applicable) are required and will appear in PubMed.

*Group Name(s): SWENAB Study Group							
***First Name and Middle Initial(s)**	***Last Name**	***Suffix (eg, Jr, III)**	Academic Degrees	Institution	Location (city, state/province, country)	Role or Contribution, eg, chair, principal investigator	Group (if more than 1 Group listed in the byline) and/or Subgroup (eg, Steering Committee)
Stellan	Håkansson		MD, Assoc Professor	The Swedish Neonatal Quality Register, Stockholm, Sweden and Department of Clinical Sciences, Pediatrics, Umeå University	Umeå, Sweden	Contributed to the design of the study and valuable insights. Participated in data collection.	
