# Supplementary material for: Antibiotic Use in Late Preterm and Full-Term Newborns
Source: JAMA Netw Open. 2024 Mar 22;7(3):e243362. doi: 10.1001/jamanetworkopen.2024.3362 (PMC10960197; doi:10.1001/jamanetworkopen.2024.3362)
Supplement: Supplement 3. — Data Sharing Statement [file jamanetwopen-e243362-s003.pdf]

Data Sharing Statement

Gyllensvärd. Antibiotic Use in Late Preterm and Full-Term Newborns. *JAMA Netw Open*. Published March 22, 2024. doi:10.1001/jamanetworkopen.2024.3362

Data
Data available: No

Additional Information
Explanation for why data not available: The data used consist of personal information that may be used to identify individuals. This is not permitted by the regulatory bodies approving this research.
